# Immune Monitoring of Mycophenolate Mofetil Activity in Healthy Volunteers Using *Ex Vivo* T Cell Function Assays

**DOI:** 10.3390/pharmaceutics15061635

**Published:** 2023-05-31

**Authors:** Aliede E. in ’t Veld, Manon A. A. Jansen, Marieke L. de Kam, Yalҫin Yavuz, Dirk Jan A. R. Moes, Kathalijne A. Oudhoff, Mariette I. E. van Poelgeest, Jacobus Burggraaf, Matthijs Moerland

**Affiliations:** 1 Centre for Human Drug Research, 2233 CL Leiden, The Netherlands; 2 Department of Surgery, Leiden University Medical Center, 2333 ZA Leiden, The Netherlands; 3 Department of Pharmacy and Clinical Toxicology, Leiden University Medical Center, 2333 ZA Leiden, The Netherlands; 4 Department of Gynecology, Leiden University Medical Center, 2333 ZA Leiden, The Netherlands; 5 Leiden Academic Centre of Drug Research, 2333 ZA Leiden, The Netherlands

**Keywords:** MMF, mycophenolate mofetil, MPA, mycophenolic acid, TDM, therapeutic drug monitoring, immunomonitoring, pharmacodynamic

## Abstract

Mycophenolate mofetil (MMF) is part of the standard immunosuppressive treatment after transplantation and usually given as “one-dose-fits-all” together with a calcineurin inhibitor (CNI). Although drug concentrations are frequently monitored, there is still a group of patients who experience side effects related to excessive or insufficient immune suppression. We therefore aimed to identify biomarkers that reflect the overall immune status of the patient and might support individualized dosing. We previously studied immune biomarkers for CNIs and aimed to investigate whether these are also suitable to monitor MMF activity. Healthy volunteers received a single dose of MMF or placebo, after which IMPDH enzymatic activity, T cell proliferation, and cytokine production were measured and compared to MPA (MMF’s active metabolite) concentration in three different matrices (plasma, peripheral blood mononuclear cells, and T cells). MPA concentrations in T cells exceeded those in PBMCs, but all intracellular concentrations correlated strongly with plasma concentrations. At clinically relevant MPA concentrations, IL-2 and IFN-γ production was mildly suppressed, while MPA T cell proliferation was strongly inhibited. Based on these data, it is expected that monitoring of T cell proliferation in MMF-treated transplantation patients may be a valid strategy to avoid excessive immune suppression.

## 1. Introduction

Mycophenolate mofetil (MMF) is an immunosuppressant that is usually combined with calcineurin inhibitors (CNIs) and corticosteroids to prevent rejection after kidney transplantation. Following oral administration, the pro-drug MMF is rapidly taken up in the upper gastrointestinal tract and converted into mycophenolic acid (MPA). MPA is an inhibitor of inosine monophosphate dehydrogenase (IMPDH), an essential enzyme for de novo guanosine synthesis. Since lymphocytes are greatly dependent on this guanosine synthesis during the S-phase of proliferation, MPA is a selective inhibitor of lymphocyte proliferation [1].

Originally, the recommended dose for MMF after renal transplantation employed a “one-dose-fits-all” approach of 2 × 1 g per day. Over recent decades, several clinical centers have introduced monitoring of MPA exposure by measuring the area under the curve (AUC0-12 h) with a limited sampling strategy (e.g., 0 h, 0.5 h, and 2 h or 0 h, 2 h, and 4 h) to prevent acute rejection in the first year after transplantation [2,3,4]. The targeted exposure is between 30 and 60 mg·h/L MPA, which is based on the data from several clinical trials where individualized dosing using limited-sampling AUCs resulted in a reduction in acute rejection [4,5,6,7,8]. It was also shown that, similar to CNIs, MMF has large variability in exposure from patient to patient [9,10]. Most side effects found in renal transplantation patients are associated with general immune suppression, such as allograft rejection, infection, diabetes, and malignancies, and occur after several years of treatment. Although these effects cannot be directly correlated with MPA exposure [11,12,13], MMF is a standard part of the immunosuppressive treatment regimen and, therefore, potentially contributes to excessive or insufficient immune suppression.

Ideally, the daily dose of immunosuppressive drugs should be adjusted to the individual needs of each transplant patient to prevent toxicity and rejection. In clinical practice, dosing is based on clinical symptoms of excessive or insufficient immune suppression (e.g., infection, toxicity) and monitoring of drug concentrations. Since the immunological response to these drugs can vary from patient to patient, monitoring of the individual patient should ideally be based on a biomarker reflecting the general immune status (level of immune suppression) rather than on drug concentrations. For CNIs, the search for these pharmacodynamics (PD)-based biomarkers has been ongoing for decades [14], but for MMF, only limited data are available. The only PD-based biomarker that has been studied in a clinical setting is the measurement of IMPDH activity, the enzyme that is directly inhibited by MPA [15]. This biomarker is specific for MPA activity and does not provide information on the effect of the other immunosuppressive drugs. Although IMPDH activity and the occurrence of rejection seem to correlate, the outcomes are highly variable.

We previously studied PD biomarkers for CNIs in healthy volunteers who received a single dose of tacrolimus or cyclosporine A. In these studies, we showed that production of IFN-γ and IL-2 in *ex vivo*-stimulated whole blood presented good biomarkers for the immunosuppressive effect of CNIs. Moreover, T cell proliferation and expression of CD154 and CD71 in T cells were also suitable to demonstrate CNI effects [16,17]. In our search for biomarkers that can reflect the overall immune status of the transplantation patient, we aimed to identify the effect of MMF treatment on these previously tested biomarkers.

Therefore, a clinical study was conducted with healthy volunteers evaluating the effect of a single dose of MMF on T cell proliferation and cytokine production. Moreover, MPA concentrations were measured in three different matrices (plasma, peripheral blood mononuclear cells, and T cells) to study the relationship with the PD biomarkers. IMPDH activity was measured to compare the selected biomarkers for MMF/MPA activity with those previously described in the literature. T cell activation was not evaluated in this study because, in our pre-clinical *in vitro* experiments this endpoint was not affected by MMF.

## 2. Materials and Methods

### 2.1. Study Design

**Figure 1:**
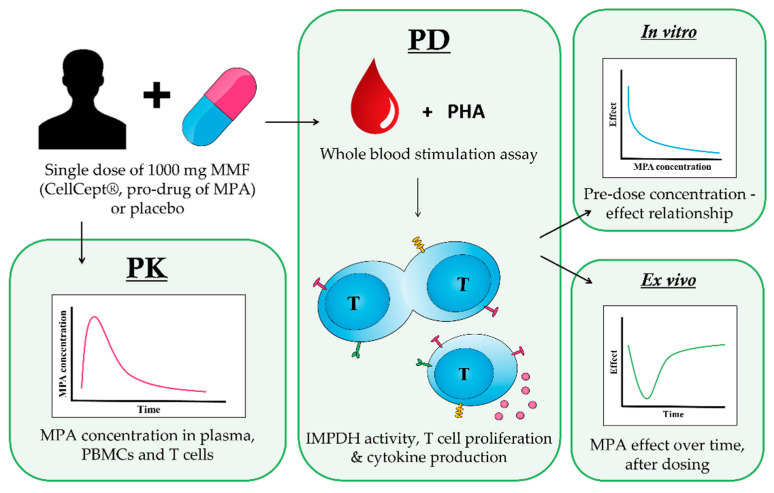
Graphical representation of the clinical study design. Twelve (12) healthy volunteers received a single dose of 1000 mg MMF (CellCept^®^, pro-drug of MPA) and four (4) healthy volunteers received a single dose of placebo. PK and PD samples were taken pre-dose (0 h) and at 0.5 h, 1 h, 2 h, 3 h, 4 h, 24 h, and 7 days post-dose. PD samples were directly used for the measurement of IMPDH activity or first stimulated with PHA to measure cytokine production and T cell proliferation. The pre-dose PD samples were used to study the *in vitro* dose–effect relationship for MPA by adding a range of concentrations of 50, 10, 2, 0.4, and 0.08 μg/L MPA to the whole-blood stimulation. Post-dose PD samples were used to study *ex vivo* effects of MPA after a single dose of MMF.

In this randomized, double-blind, placebo-controlled study, sixteen healthy volunteers were enrolled. Healthy male or female subjects 18–55 years of age who gave written informed consent and did not have any disease associated with immune system impairment were included. All subjects received a single oral dose of 1000 mg MMF (CellCept^®^, Roche Pharma AG, Grenzach Wyhlen, Germany), which is the recommended daily dose for renal transplant patients receiving MMF as maintenance immunosuppressive therapy (1000 mg CellCept^®^ twice daily). A total of twelve subjects received active treatment and four subjects received the placebo. Both PK and PD samples were taken pre-dose (0 h) and at 0.5 h, 1 h, 2 h, 3 h, 4 h, 24 h, and 7 days post-dose (Figure 1). This study was approved by the “Medisch Ethische Toetsingscommissie van de Stichting Beoordeling Ethiek Biomedisch Onderzoek” (Assen, The Netherlands) on 30 April 2019 and is registered in the International Clinical Trials Registry Platform (ICTRP) under study number NL7804. The study was performed in compliance with the Dutch laws on drug research in humans.

### 2.2. Plasma and Intracellular PK

Concentrations of MPA, the active form of the pro-drug MMF, were measured in plasma, peripheral blood mononuclear cells (PBMCs), and T cells. Samples were processed as described previously [17], and the isolated cells were frozen in PBS until analysis. The quantification of MPA in plasma, PBMCs, and T cell samples was performed by the ISO15189-accredited Clinical Pharmaceutical Laboratory of the Department of Clinical Pharmacy and Toxicology, Leiden University Medical Center, the Netherlands.

MPA concentration in plasma was quantified using a previously validated LC-MS/MS assay [18]. A new method was developed for the quantification of intracellular concentrations in PBMCs and T cells, which was performed in a similar way as for the measurement of intracellular cyclosporine A concentrations [17]. In short, the calibration standards and quality controls used were mycophenolic acid (Alsachim, Illkirch-Graffenstaden, France) and mycophenolic acid-D3-C13 (Alsachim, France) prepared in acetonitrile (10 mg/L). Calibration standards of 0.1, 0.2, 0.5, 1, 5, 10, 20, 50, and 100 ug/L and QCs of 0.5, 5, and 50 ug/L were diluted in MPA-free PBMCs and used in every analytical run. Before measurement, 100 μL of sample was mixed with 20 μL of internal standard solution (20 μg/L) and vortexed for 15 min. Further sample processing and analysis were performed as previously described for intracellular cyclosporine A measurement, with the following mass transitions for multiple reaction monitoring acquisition (*m*/*z*): mycophenolic acid 321.1→207.0, mycophenolic acid-D3-C13 325.1→211.0. All analytical validation parameters were in accordance with the EMA bioanalytical method validation guideline.

### 2.3. Cytokine Production and T Cell Proliferation

For measurement of cytokine production, whole blood was stimulated for 24 h with 10 μg/mL phytohemagglutinin (PHA) (Sigma Aldrich, St. Louis, MO, USA), as described previously [17]. At the pre-dose time point, the *in vitro* MPA concentration–effect relationship for each individual subject was studied by incubating whole-blood samples with concentrations of 50, 10, 2, 0.4, and 0.08 μg/L MPA (Sigma Aldrich). To study the immunosuppressive effect of MPA *ex vivo*, post-dose whole-blood samples were incubated with PHA only. IFN-γ and IL-2 concentrations were measured with the Meso Scale Discovery Vplex-2 method by Ardena Bioanalytical Laboratory in Assen, the Netherlands.

T cell proliferation was measured in the same way as in our previous study [17] using an EdU kit (Thermo Fisher Scientific, (Waltham, MA, USA). A MACSQuant 16 analyzer (Miltenyi Biotec, Bergisch Gladbach, Germany) was used for flow cytometry analysis of EdU incorporation. T cell proliferation was expressed as the percentage of EdU-positive cells relative to the total number of T cells.

### 2.4. IMPDH Enzyme Activity

Preceding the start of the clinical study, the *in vitro* relationship between MPA concentration and IMPDH activity was evaluated in fresh whole blood from three healthy donors. Sodium-heparinized whole-blood samples were incubated for 1 h at 37 °C with 5% CO_2_ with a range of MPA concentrations (50, 10, 2, 0.4, 0.08, and 0 μg/L). After incubation, PBMCs were isolated from lithium-heparinized whole blood using Lymphoprep and SepMate tubes (Stemcell Technologies Inc., Vancouver, Canada). After washing, the cells were frozen at −80 °C in distilled water. Furthermore, *ex vivo* IMPDH activity was monitored in the clinical study in freshly isolated and stored PBMCs that did not undergo incubation.

Final analysis of IMPDH enzymatic activity was performed by Ardena Bioanalytical Laboratory in Assen, the Netherlands, using liquid chromatography with tandem mass spectrometry (LC-MS/MS). The PBMC lysate was incubated at +37 °C for 3 h in the presence of the IMPDH substrate inosine-5′-monophosphate (IMP) and NAD+. The amount of xanthosine-5′-monophosphase (XMP) was measured using LC-MS/MS. To express the IMPDH activity in μmol XMP/min/mg protein, the protein content of the PBMC sample lysates was measured using the Pierce BCA Protein Assay Kit (Thermo Fisher Scientific).

### 2.5. Data Analysis

**Figure A1:**
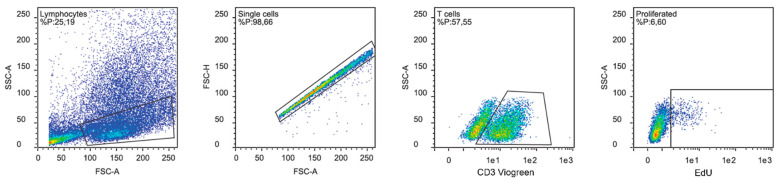
Gating strategy for T cell proliferation.

Flow cytometry data analysis was performed with Flowlogic software version 7.3 (Inivai Technologies, Mentone VIC, Australia), the gating strategy of which is shown in Appendix A, Figure A1. Data for all plots are presented as mean values ± standard deviation (SD). No formal power analysis was performed given the explorative character of the study. For that reason, no statistical analysis was applied to discriminate between active and placebo treatment. Repeated-measures correlation [19] (rmcorr) was used for determining the common within-individual association for repeated measures assessed at multiple time points for multiple individuals. Repeated-measures correlation was conducted using the rmcorr R package [20,21].

## 3. Results

### 3.1. Subject Characteristics and Safety

A total of 12 subjects received a single dose of CellCept (MMF) and 4 subjects received the placebo. The baseline characteristics of the 16 healthy volunteers are summarized in Table 1. A total of five adverse events (AEs) occurred during the study, which were fatigue and decreased platelet count. The AEs were all characterized as mild and only occurred in CellCept-treated subjects.

### 3.2. Plasma and Intracellular Pharmacokinetics

**Figure 2:**
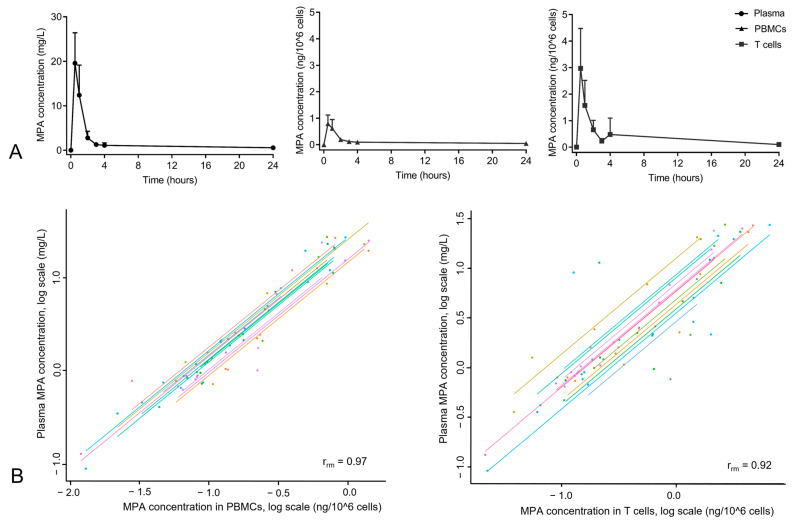
(**A**) Mean concentration of MPA in plasma, PBMCs, and T cells after a single dose of 1000 mg MMF. Samples were taken at 0 h, 0.5 h, 1 h, 2 h, 3 h, 4 h, and 24 h. (**B**) Repeated-measures correlation for MPA in plasma vs. PBMCs and plasma vs. T cells. Each subject is represented by a different color, and pair concentration at each time point is indicated with a dot. Repeated-measures correlation (rrm) was calculated, and the correlation coefficient is shown in the plots.

In Figure 2A, the MPA plasma concentration is shown. The highest plasma concentration was observed at 0.5 h post-dose (19.6 ± 6.8 mg/L), after which it strongly decreased to a concentration of 1.1 mg/L (± 0.97 mg/L) at 4 h post-dose. Intracellular MPA concentrations in PBMCs and T cells showed a comparable PK profile to the plasma concentration. The peak concentration in T cells was 3.75 times higher compared to the peak concentration in PBMCs, indicating a preferential uptake of MPA by T cells in the circulation, provided that different isolation procedures were not responsible for this difference.

It has been stated that most of the administered MMF is metabolized into the inactive mycophenolic acid glucuronide (MPAG) and excreted in urine. Part of the MPAG is excreted in bile, after which it is back-converted into MPA and reabsorbed in the colon, leading to an increase in MPA plasma concentration [22]. In our data, this enterohepatic recirculation was clearly visible in the intracellular MPA concentrations in T cells, where a second peak at 4 h post-dose was found. Although not for all subjects, this second peak in MPA concentration was also visible in PBMCs (in PBMCs for three subjects and T cell concentrations for six subjects). For none of the subjects could a second peak in plasma concentration be detected.

Finally, the repeated-measures correlations between MPA plasma concentrations and intracellular concentrations are shown in Figure 2B. Intracellular MPA concentrations in both PBMCs and T cells showed strong repeated-measures correlations with plasma concentrations that were measured at the same time points (r_rm_ of 0.97 and 0.92, respectively), indicating that these intracellular PK parameters provide us with similar information as the plasma concentrations.

### 3.3. IMPDH Enzymatic Activity

**Figure 3:**
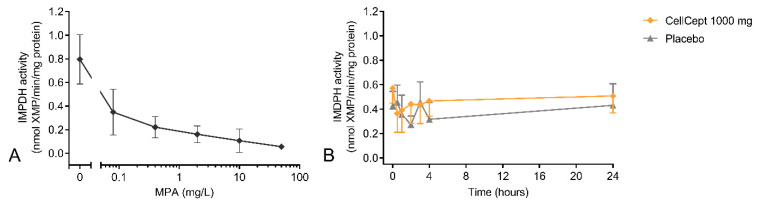
(**A**) *In vitro* and (**B**) *ex vivo* MPA effects on inosine-5′-monophosphate dehydrogenase (IMPDH) enzymatic activity. *In vitro* samples were incubated with a range of concentrations of MPA (50, 10, 2, 0.4, and 0.08 mg/L). *Ex vivo* samples were taken before and 0.5 h, 1 h, 2 h, 3 h, 4 h, and 24 h after subjects were dosed with MMF.

Whole-blood incubations with a range of concentrations of MPA resulted in a strong inhibitory *in vitro* effect from MPA on inosine-5′-monophosphate dehydrogenase (IMPDH) activity (Figure 3). At a concentration of 0.08 mg/L MPA, IMPDH activity was inhibited by more than 50%, and a maximum inhibition of 93% was reached at a concentration of 50 mg/L. The *in vitro* dose–response relationship, which was studied in three donors, supported the inclusion of the IMDPH assay in the clinical study. The maximum *ex vivo* MPA effect was an inhibition of 28% at 30 min post-dose and could not be discriminated from the IMPDH activity in the placebo group at the same time point. Moreover, the variability in the maximum *ex vivo* MPA effect (CV of 82%) indicates there was strong intersubject variability. At 30 min post-dose, a plasma concentration of 19.6 mg/L MPA was measured, which was expected to result in an inhibition of ~90% IMPDH activity based on the *in vitro* dose–response relationship, indicating that *in vitro* MPA effects were not predictive of the *ex vivo* MPA effect.

### 3.4. T Cell Proliferation

**Figure 4:**
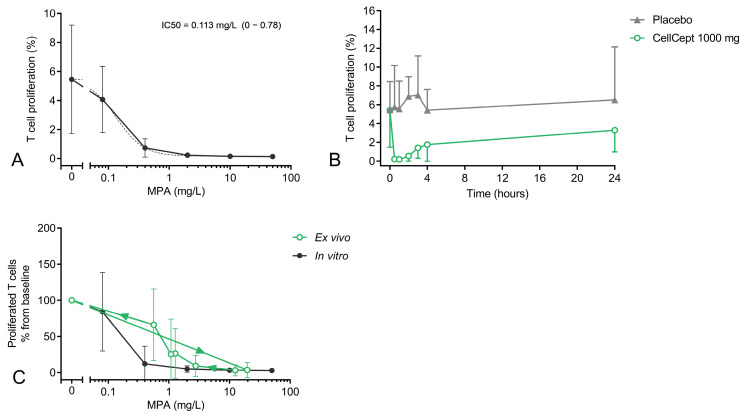
(**A**) *In vitro* and (**B**) *ex vivo* MPA effects on T cell proliferation. *In vitro* samples were incubated with a range of MPA concentrations (50, 10, 2, 0.4, and 0.08 mg/L). The absolute data points (± SD, solid line) and logistic regression model (dotted line) are shown. *Ex vivo* samples were taken before and 0.5 h, 1 h, 2 h, 3 h, 4 h, and 24 h after the subjects were dosed with MMF. (**C**) An overlay of the *in vitro* and *ex vivo* MPA effects on T cell proliferation expressed as percentage difference from baseline. Arrows indicate the time course of the samples.

In Figure 4, the *in vitro* and *ex vivo* effects of MPA on PHA-induced T cell proliferation are shown. With an IC50 of 0.113 mg/L, a strong dose–effect relationship was found *in vitro* (Figure 4A). Although the IC50 varied widely from subject to subject (95% CI of 0–0.78), nearly all subjects already reached the maximum inhibitory effect on T cell proliferation (inhibition of 95 ± 5%) at a concentration of 2 mg/L MPA.

**Figure A2:**
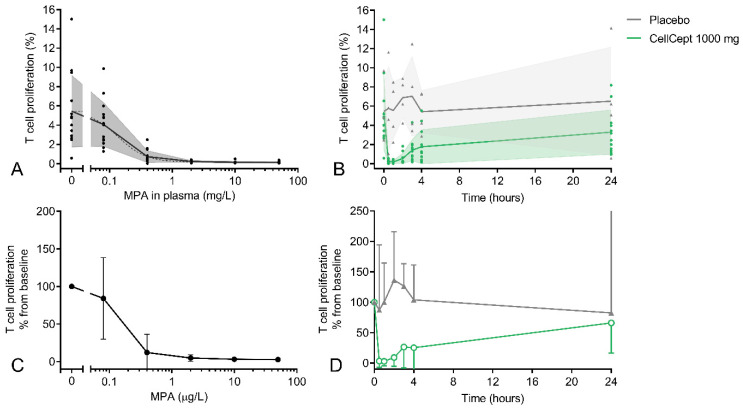
*In vitro* (**A**,**C**) and *ex vivo* (**B**,**D**) MPA effects on T cell proliferation. Data are expressed as percentage of T cell proliferation with dots representing the individual data points (**A**,**B**) and as percentage change from baseline (**C**,**D**). Grey triangles represent placebo subjects and green circles represent MMF-treated subjects.

Furthermore, a strong *ex vivo* MPA effect on T cell proliferation was observed compared to placebo (Figure 4B). At 30 min post-dose, the proliferation was completely inhibited (97% ± 11%), and even after 24 h, the proliferation was still reduced compared to baseline. However, there was variation in proliferation between subjects. To clarify the *in vitro* MPA concentration–effect relationship in relation to *ex vivo* MPA activity (measured post-dose), the overlay of the *in vitro* and *ex vivo* MPA effects on T cell proliferation is shown in Figure 4C. While an *in vitro* MPA concentration of 0.4 mg/L already resulted in an 89% inhibition of T cell proliferation, substantially higher MPA exposure was required to reach comparable T cell proliferation inhibition *ex vivo*. At the 3 h, 4 h, and 24 h time points, plasma concentrations of 1.3, 1.1, and 0.6 mg/L MPA were found, which resulted in inhibition of T cell proliferation of 74%, 75%, and 34%, respectively (Appendix A, Figure A2). Although the variability in T cell proliferation between subjects was high at these time points (CV of 130%, 191%, and 75%, respectively), the discrepancy between *ex vivo* and in vivo was visible for all individual subjects.

### 3.5. Cytokine Production

**Figure 5:**
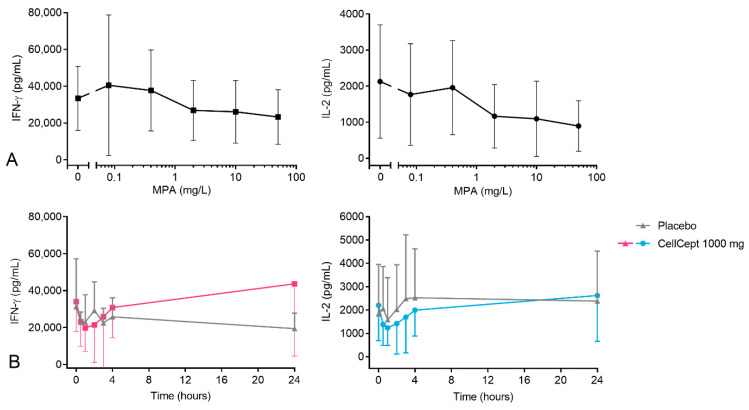
(**A**) *In vitro* and (**B**) *ex vivo* MPA effects on IFN-γ and IL-2 production. *In vitro* samples were incubated with a range of MPA concentrations (50, 10, 2, 0.4, and 0.08 mg/L). *Ex vivo* samples were taken before and 0.5 h, 1 h, 2 h, 3 h, 4 h, and 24 h after the subjects were dosed with MMF.

The last PD biomarkers studied were represented by PHA-induced production of IFN-γ and IL-2. Incubation of pre-dose whole-blood samples with a range of MPA concentrations did not result in a strong *in vitro* effect on cytokine production (Figure 5A). For IL-2 production, inhibition at the higher MPA concentrations was found (inhibition of 53% and 70% at MPA concentrations of 10 and 50 mg/L), but the effect size was variable between subjects (inter-individual CV of 64% and 54%, respectively). For IFN-γ production, the *in vitro* MPA effect was less pronounced, with inhibition of 30% and 50% (inter-individual CV of 67% and 43%, respectively) at the highest MPA concentrations.

**Figure A3:**
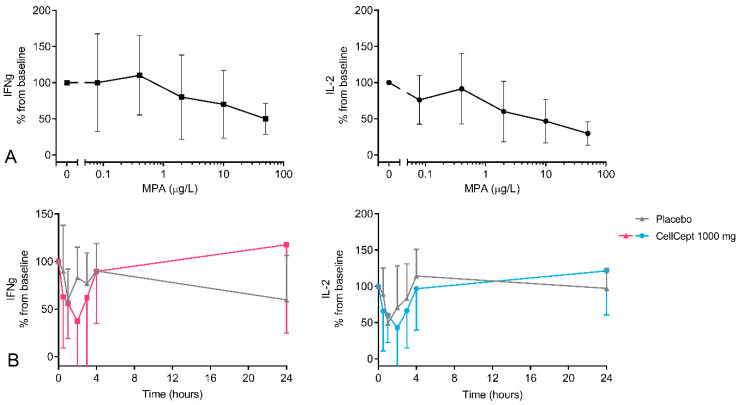
*In vitro* (**A**) and *ex vivo* (**B**) MPA effects on IFN-γ and IL-2 production expressed as percentage change from baseline.

After MMF intake, a decrease in the PHA-induced production of IFN-γ and IL-2 was visible in the first 4 h post-dose (Figure 5B). In the placebo-treated subjects, however, a comparable decrease in cytokine production was found, making it impossible to discriminate between the placebo- and MMF-treated subjects. Change-from-baseline figures can be found in Appendix A, Figure A3.

## 4. Discussion

Ideally, the daily dose of immunosuppressive drugs prescribed to renal transplant patients (e.g., tacrolimus, MMF, and prednisolone) should be adjusted to the individual needs of the patient. As these patients will use immunosuppressive drugs for the rest of their lives, it is important to attain an optimal balance between over- and undersuppression of immunity. Individualized therapy could be advanced by the availability of biomarkers that reflect the immunosuppressive state of individual patients rather than dosing based on drug concentrations or the occurrence of side effects. In the search for such PD biomarkers, we previously selected functional T cell assays for monitoring of the immunosuppressive effects of cyclosporine A and tacrolimus [16,17]. In the current study, we investigated if these biomarkers are also suitable to monitor the immunosuppressive effect of MMF and examined their relationship with drug concentrations.

Twelve healthy volunteers received a single oral dose of 1000 mg MMF, after which MPA (the active form of MMF) concentrations were measured in plasma, PBMCs, and T cells. With a peak concentration of 19.6 mg/L MPA at 0.5 h and a concentration of 1.1 mg/L MPA at 4 h, the plasma PK profile was similar to what has been previously reported for healthy volunteers [23], with a slightly higher exposure than that found in stable renal transplantation patients (8–15 mg/L) [24,25]. Little is known about the relationship between PBMC and plasma MPA concentrations. While in early renal transplantation patients, there was no correlation between plasma and PBMC MPA levels at C0 (pre-dose), at later time points, a strong correlation at 1.5 and 3.5 h post-dose was found [26]. Although, in our study, the method of measuring intracellular drug concentrations was different, we found similar results. A strong repeated-measures correlation between plasma concentrations and the MPA concentrations inside the target cell (e.g., PBMCs and T cells) was found. Based on our data from healthy volunteers, there is no added value in measuring intracellular MPA concentrations rather than plasma concentrations.

Interestingly, the MPA concentrations in T cells were higher than those in PBMCs at the same time points, which contrasts with what we previously reported for intracellular tacrolimus and cyclosporine A concentrations [16,17]. T cells are the largest cell population present in PBMCs, suggesting that T cells take up more MPA into the cell compared to all other cell populations that are present in PBMCs (i.e., monocytes, B cells, NK cells, dendritic cells). This would be beneficial as lymphocytes are the targeted population for post-transplant immunosuppressants and are primarily affected by MPA. This is the first study in which MPA concentrations inside T cells have been measured. It should be noted that the methods of MPA measurement for PBMCs and T cells were identical, but the methods of cell isolation for these populations were different. Therefore, there is a possibility that the difference in PK between PBMCs and T cells was also affected by the technical differences.

The PD marker for MMF that is most frequently described in the literature is IMPDH enzymatic activity [15]. IMPDH catalyzes the oxidation of inosine monophosphate (IMP) to xanthosine monophosphate (XMP), which is an essential step in DNA synthesis of proliferating lymphocytes. Since MPA is an IMPDH inhibitor, enzymatic IMDH activity is the biomarker that is closest to the drug target. A strong *in vitro* concentration–response relationship with a maximum inhibition of 96% for IMPDH activity was found. Based on these *in vitro* data, the plasma concentration of 19.6 mg/L MPA, observed at 0.5 h after MMF treatment, was expected to result in an inhibition of ~90% for IMPDH activity. However, no substantial *ex vivo* effect from MMF treatment on IMPDH activity was observed, indicating a large discrepancy between *in vitro* and *ex vivo* MPA effects for which we currently do not have an explanation. The method used to measure IMPDH activity was similar to what has been previously reported in the literature and was successful in demonstrating the inhibitory effect of MMF in renal transplantation patients, with a maximum inhibition of 75% at MPA plasma concentrations of ~7 mg/L [27]. We saw a comparable effect in the *in vitro* dose–response relationship but to a much lesser extent in the *ex vivo* data. Looking at the IMPDH activity in the placebo group of the study, there was large variability in IMPDH activity over time. This has also been described in the literature and is the reason that studies are currently more focused on measurement of IMPDH activity in erythrocytes than on using PBMCs [15,28]. Moreover, although IMPDH activity is an interesting readout measure to demonstrate direct MPA effects, it does not provide information about the activity of the overall immune response.

T cell proliferation is an immune biomarker that is directly affected by IMPDH activity, being more distal from the drug target. Of all the PD readout measures that were studied, proliferation had the strongest *in vitro* concentration–effect relationship, with an IC50 of 0.11 mg/L MPA. Interestingly, the concentration at which maximum inhibition of T cell proliferation was reached *in vitro* varied from subject to subject. While some subjects showed complete inhibition of T cell proliferation at 0.4 mg/L MPA, in other subjects, only 40% inhibition was found at this MPA concentration. These observations were consistent with the *ex vivo* MPA effect at 3–4 h post-dose, when lower plasma concentrations were measured. At these time points, intrasubject variability was higher than at the other time points (CVs of 78–100%), indicating that the concentration at which 100% inhibition was reached varied from subject to subject. In clinical practice, a target AUC_0–12 h_ of 30–60 mg x /L is recommended because this has been associated with lower occurrence of allograft rejection [4]. On average, these AUC reference values correspond roughly to a trough concentration (C0) of ~2 mg/L [29,30]. At this concentration, both our *in vitro* and *ex vivo* data showed >90% suppression of T cell proliferation, indicating that, with the current AUC monitoring strategy, T cell proliferation in renal transplantation patients is most likely always completely suppressed. However, we did identify reasonable intersubject variability in the concentration at which maximal T cell inhibition was found. If this is also true in renal transplantation patients, it might be more relevant to find the lowest AUC at which an individual patient reaches maximum inhibition of T cell proliferation rather than maintaining the same AUC reference values for all patients.

Recognition of MHC/antigen complexes by T cell receptors and CD3 results in T cell activation and subsequent pro-inflammatory gene expression, including IL-2. By binding its receptor (CD25), IL-2 induces differentiation and proliferation of cytotoxic T cells and T helper cells, resulting in the production of more IL-2 and other pro-inflammatory cytokines, such as IFN-γ [31]. PHA-induced cytokine production, as measured in our study, therefore did not directly reflect IMPDH activity but was rather a more general measure of T cell responsiveness. While tacrolimus and cyclosporine A significantly impact IL-2 and IFN-γ production [16,17], no such effect was observed for the MPA exposures evaluated in this study *in vitro* or *ex vivo*. *In vitro*, MPA concentration-dependently inhibited IL-2 production with a maximum inhibition of 70%, while for IFN-γ production, the highest concentration of MPA resulted in an inhibition of 53%. This rather limited effect could be explained by the fact that tacrolimus and cyclosporine A directly act on T cell activation via suppression of nuclear factor of activated T cells (NFAT), while MPA only affects T cell proliferation but not activation. T cells present in the whole-blood culture can probably still produce cytokines, but the MPA-mediated reduction in proliferation resulted in a smaller number of T cells present in the culture. Despite the moderate inhibitory effect of MPA *in vitro*, no *ex vivo* MPA effect on cytokine production was seen. A dip in cytokine production was visible at 1 h post-dose in both the MMF and the placebo groups, potentially caused by the diurnal rhythmicity in circulating T cell numbers [32].

T cell proliferation and PHA-induced IL-2 and IFN-γ production have previously been shown to be effective biomarkers in demonstrating the immunosuppressive effects of calcineurin inhibitors. Since renal transplantation patients often receive the combination of a calcineurin inhibitor with MMF, we aimed to investigate whether these biomarkers are also suitable for monitoring MMF treatment effects. We substantiated this biomarker panel with IMPDH activity, reflecting proximal MPA activity. PHA-driven production of IL-2 and IFN-γ was only mildly affected by MPA at clinically relevant exposures, and we could not discriminate between placebo- and MMF-treated subjects in terms of IMPDH enzymatic activity. However, we showed that PHA-induced T cell proliferation is an excellent measure to monitor MPA activity. The only limitation of this study was the small subject population, which prevented formal statistical and PK/PD analyses from being performed and resulted in high intersubject variability for the readout measures.

In conclusion, based on the observed MPA plasma concentrations in a renal transplantation setting [33] and our *in vitro* and *ex vivo* MPA effects, it is expected that renal transplantation patients will always have completely inhibited T cell proliferation in conventional clinical MMF regimens, as well as at MPA trough levels. The MPA concentration at which maximum T cell inhibition was reached varied from subject to subject, implying that MMF dose reduction based on measurement of T cell proliferation in transplant patients may be a valid future strategy to avoid oversuppression of the immune system with MMF. Before this biomarker can be used in transplantation patients, however, we need to further validate these readout measures in a clinical setting. Since MMF is usually given in combination with CNIs, a follow-up study with renal transplantation patients to investigate the relationship between MMF and CNI dose, T cell proliferation, and clinical outcome would be valuable.

## Figures and Tables

**Table 1:** Baseline subject characteristics.

Subject Characteristics	1000 mg CellCept (*n* = 12)	Placebo (*n* = 4)
Age (range)	24.8 (18–39)	27.2 (18–46)
Gender (female/male)	5/7	3/1
BMI (kg/m^2^), mean (range)	24.41 (18.7–28.9)	21.85 (20.1–24.3)

## Data Availability

The data presented in this study are available on request from the corresponding author.
